# Sensitive and highly resolved identification of RNA-protein interaction sites in PAR-CLIP data

**DOI:** 10.1186/s12859-015-0470-y

**Published:** 2015-02-01

**Authors:** Federico Comoglio, Cem Sievers, Renato Paro

**Affiliations:** 1 Department of Biosystems Science and Engineering, Swiss Federal Institute of Technology Zurich, Mattenstrasse 26, Basel, 4058 Switzerland; 2 Department of Pathology and Center for Cancer Research, Massachusetts General Hospital and Harvard Medical School, Boston, USA; 3 Broad Institute of MIT and Harvard, Cambridge, USA; 4 Faculty of Science, University of Basel, Klingelbergstrasse 504056, Basel, Switzerland

**Keywords:** PAR-CLIP, RNA, RNA binding proteins, Bayesian statistics

## Abstract

**Background:**

PAR-CLIP is a recently developed Next Generation Sequencing-based method enabling transcriptome-wide identification of interaction sites between RNA and RNA-binding proteins. The PAR-CLIP procedure induces specific base transitions that originate from sites of RNA-protein interactions and can therefore guide the identification of binding sites. However, additional sources of transitions, such as cell type-specific SNPs and sequencing errors, challenge the inference of binding sites and suitable statistical approaches are crucial to control false discovery rates. In addition, a highly resolved delineation of binding sites followed by an extensive downstream analysis is necessary for a comprehensive characterization of the protein binding preferences and the subsequent design of validation experiments.

**Results:**

We present a statistical and computational framework for PAR-CLIP data analysis. We developed a sensitive transition-centered algorithm specifically designed to resolve protein binding sites at high resolution in PAR-CLIP data. Our method employes a Bayesian network approach to associate posterior log-odds with the observed transitions, providing an overall quantification of the confidence in RNA-protein interaction. We use published PAR-CLIP data to demonstrate the advantages of our approach, which compares favorably with alternative algorithms. Lastly, by integrating RNA-Seq data we compute conservative experimentally-based false discovery rates of our method and demonstrate the high precision of our strategy.

**Conclusions:**

Our method is implemented in the R package wavClusteR 2.0. The package is distributed under the GPL-2 license and is available from BioConductor at http://www.bioconductor.org/packages/devel/bioc/html/wavClusteR.html.

**Electronic supplementary material:**

The online version of this article (doi:10.1186/s12859-015-0470-y) contains supplementary material, which is available to authorized users.

## Background

RNA-binding proteins (RBPs) play a fundamental role in virtually all aspects of RNA metabolism, including the regulation of RNA localization, stability, translation or degradation [1]. These proteins extensively contribute to the control of gene expression by regulating the life cycle of microRNAs, where the RBP-RNA interaction is mediated by specific RNA sequence motifs or secondary structures [2]. Interestingly, recent studies showed that deregulation of RBP expression or mutation of cognate binding sites are causally related to several human diseases including cancer [3-6]. Many of these studies have been made possible by the development of new methods mapping interaction sites in a comprehensive and systematic manner [7]. Particularly, the Photo-Activatable Ribonucleoside-enhanced CrossLinking and ImmunoPrecipitation (PAR-CLIP) method made it possible to identify highly specific RBP-RNA interactions by generating a distinct imprint in the bound RNA [8-10]. In this method, cells are cultured with a ribonucleoside analogue, e.g. 4-thiouridine (4SU), which becomes incorporated into nascent RNA molecules. Then, *in vivo* UV crosslinking at a specific wavelength is performed to stabilize the RNA-RBP interaction, resulting in a covalently linked RNA-RBP complex. Next, the complex is isolated, the protein digested, the RNA molecules recovered and reverse transcribed to cDNA. Next-Generation Sequencing is then used to determine the identity of these molecules.

Importantly, the reverse transcription induces specific base transitions at the original cross-linked sites, which can be used to identify high-confidence RBP-RNA binding interactions in PAR-CLIP data [11]. Based on the induction of transitions, different strategies have been developed for the identification RNA-RBP interactions in PAR-CLIP data. CLIPZ [12], a widely adopted method for PAR-CLIP data analysis, ranks protein binding sites, referred to as clusters, based on their total number of observed transitions. PARalyzer [13], in contrast, utilizes transitions to fit a Gaussian kernel density estimate classifier in order to discriminate signal from noise at interaction sites with the aim to infer the protein binding sites. The cluster boundaries are determined by extending the interaction sites using a fixed threshold on the coverage or by applying an arbitrary window size. PIPE-CLIP [14], a very recent tool designed for CLIP-seq data analysis, employs a binomial model and performs comparably to PARalyzer in identifying binding sites in PAR-CLIP data. However, these methods fall short on important aspects of PAR-CLIP data analysis. (i) As experimental validation of RNA-RBP interactions is laborious and only feasible on small scale, statistically rigorous approaches are needed to rank clusters and identify high-confidence subsets amenable to experimental testing. (ii) PAR-CLIP data allows for a highly resolved identification of the RBP binding sites. However, to delineate cluster boundaries accurately, a sensitive peak caller tailored to this problem is needed. (iii) Not every observed transition is induced by cross-linking, i.e. by PAR-CLIP. Rather, sequencing errors, RNA contaminants and cell type-specific SNPs represent additional sources of transitions which can lead to the detection of a considerable number of false positives [11]. Attempts to limit the false discovery rate (FDR) by requiring a minimum number of interaction sites per cluster, as recommended by [13,15], can largely reduce sensitivity as it will inevitably miss all true clusters containing less interaction sites. In fact, given that the nucleotide composition of protein binding sites can greatly vary, clusters exhibiting a few PAR-CLIP induced transitions can still correspond to *bona fide* interaction sites.

In this work, we specifically address the three points outlined above. We introduce a Bayesian model to identify PAR-CLIP induced high-confidence transitions extending our recent work in [11]. We detail a new, coverage-based algorithm for the identification of cluster boundaries termed Mini-Rank Norm (MRN) and show that it substantially improves resolution of binding sites over other methods. We test our algorithm on published data and compare its performance with PARalyzer. We demonstrate that wavClusteR outperforms alternative algorithms both in detection and resolution of clusters. By using a transition frequency-based strategy our method overcomes the reduction in sensitivity and specificity which characterizes hard thresholding approaches such as PARalyzer. Lastly, we evaluate the performance of our algorithm by integrating matched RNA-Seq data to compute conservative FDR estimates, confirming that high-confidence transitions identified by our approach are PAR-CLIP specific.

## Methods

### Model

Let *i* be a genomic position spanned by a number of reads after the short read alignment. The relative substitution frequency (RSF) *x* at position *i* is the ratio between the number of base substitutions *y* within the reads (e.g. T → C) aligned at *i* relative to the total coverage *z* at the site, and can be interpreted as an estimate of the corresponding transition probability. We recently introduced a non-parametric, two-component mixture model to discriminate PAR-CLIP-specific from non-experimentally-induced transitions [11]. In our model, the first and second component represent non-experimental and PAR-CLIP-induced transitions, respectively.

**Figure 1:**
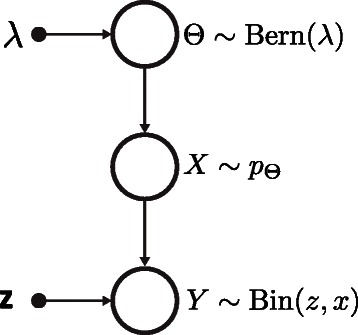
**Graphical representation of the Bayesian network employed to discriminate PAR-CLIP-specific from extrinsic transitions.** The data generating process is modeled hierarchically, where i) *Θ* encodes the unobserved source of the transition (PAR-CLIP-specific or -independent), ii) *X* represents the relative substitution frequency and iii) *Y* is the number of observed transitions at the position, which exhibits a coverage of *z* reads.

Here, we developed a model that integrates information over the entire RSF range. For this purpose, we consider a Bayesian network representation of our mixture model (Figure 1) corresponding to a chain of three random variables (*Θ*,*X*,*Y*). Here *Θ*∈{1,2} encodes the source of transition (non-experimental [ *Θ*=1] or experimental [ *Θ*=2]), *X*∈(0,1] represents a relative substitution frequency (RSF) value and *Y* is the number of observed transitions at a given position. According to this model all observed substitutions are thought to be generated as follows. First, a binary random number *Θ* is drawn from a Bernoulli distribution Bern(*λ*) with *p*(*Θ*=1)=*λ*. The value of *Θ* determines the component used to sample the base substitution probability *x*. Second, the number of observed transitions *Y* is obtained from a binomial distribution Bin(*z*,*x*), where the sample size *z* corresponds to the total number of aligned reads at a given position. According to our model, *p*(*Y*,*X*,*Θ*) factorizes as:

$$p(Y, X, \Theta) = p(Y | X) p(X | \Theta) p(\Theta). $$

Therefore, the posterior probability that a given number of transitions was induced by either source can be computed as:

$$p(\Theta=\theta | Y) = \frac{p(Y,\theta)}{p(Y)}=\frac{{\int_{0}^{1}} p(Y | x) p(x | \theta) p(\theta) dx }{p(Y)}. $$

The resulting posterior probability marginalizes out *X* and thereby integrates information over the entire RSF range. Using *p*(*Θ*=*θ*|*Y*), we then compute the log-odds ratio for each transition as log(*p*(*Θ*=2|*Y*)/*p*(*Θ*=1|*Y*) and define the relative log-odds ratio for a cluster as the sum of all log-odds within a cluster, normalized to the total number of bases susceptible to cross-linking.

### Cluster boundaries identification

Let ${\mathcal {C}}(i)$ be the coverage at position *i* and ${\mathcal {C}}(i,j)$ be the sequence of coverage values $({\mathcal {C}}(i), {\mathcal {C}}(i+1), \ldots, {\mathcal {C}}(j)), i\leq j$. Similarly, let ${\mathcal {S}}(i)$, ${\mathcal {E}}(i)$ be the positive and negative differences in the coverage function, i.e. the number of read alignments starting or ending at position *i*, respectively, and ${\mathcal {S}}(i,j) = ({\mathcal {S}}(i), {\mathcal {S}}(i+1), \ldots, {\mathcal {S}}(j))$, ${\mathcal {E}}(i,j) = ({\mathcal {E}}(i), {\mathcal {E}}(i+1), \ldots, {\mathcal {E}}(j))$, *i*≤*j* be the extended notation to intervals. We then consider the set  of all genomic positions corresponding to high-confidence transitions (hcTs) of a given type (e.g. T →C). In the following paragraphs, we detail the steps performed by the MRN algorithm.

#### Estimate local background threshold

**Figure 2:**
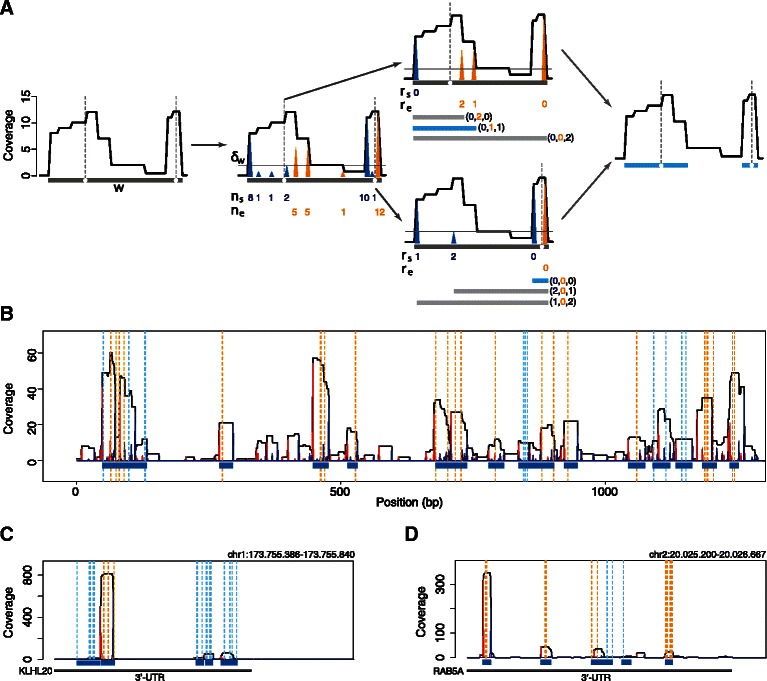
**Outline of the MRN algorithm and examples.** **(A)** A non-zero coverage window *w* containing two high-confidence transitions (hcTs, denoted by white circles and dashed gray lines) located in close proximity but marking two distinct protein binding sites is shown (gray rectangle). In this window, the observed total coverage of aligned PAR-CLIP reads is indicated by the solid black line, which exhibits two proximal peaks corresponding to the interaction sites. The MRN algorithm aims at resolving these binding sites by discriminating binding-dependent coverage fluctuations from noise and by subsequently using geometric properties of RBP binding sites to refine the cluster boundaries. First, all coverage fluctuations, i.e. positive and negative coverage differences (blue and orange triangles, respectively, with heights proportional to the magnitude of the coverage fluctuations) within *w* are computed and stored in the two vectors *n*_*s*_ and *n*_*e*_, respectively. These values are then used to learn a local threshold *δ*_*w*_ (see Methods) that is applied to remove noise from the coverage function. Coverage fluctuations smaller than *δ*_*w*_ (solid gray line) are discarded. Next, each hcT is processed separately. The values of all retained positive and negative coverage differences localizing upstream and downstream to the analyzed transition, respectively, are ranked. Rankings are stored in the rank vectors *r*_*s*_ and *r*_*e*_. Finally, all putative cluster boundaries (rectangles) are identified and their length is ranked. Each candidate cluster, represented by a rank vector summarizing coverage and cluster length rankings (e.g. (0,2,0)), is then evaluated and the optimal cluster (light blue) is identified (see Methods). **(B)** Clusters (blue rectangles) identified by the MRN algorithm within a complex coverage region of length 1.6 kb of chromosome 10, MOV10 data set. Positive and negative coverage differences are shown in red and blue, respectively. hcTs are indicated by vertical dashed lines. Cyan lines correspond to hcTs that solely localize within clusters identified by the MRN algorithm. Clusters identified using the CWT-based algorithm do not contain these sites. **(C)** AGO2 clusters identified within the 3’-UTR of the KLHL20 transcript. Each cluster contains ≥1 microRNA seed sequences of microRNAs expressed in HEK293 cells. The color scheme is the same as in B. **(D)** Same as C, but for the 3’-UTR of the RAB5A transcript.

For each $i_{t} \in {\mathcal {T}}$, we consider the largest non-zero coverage window *w* containing *i*_*t*_ and compute all putative cluster start $C_{s}=({\mathcal {S}}(i) \geq \delta)_{i \in w: i\leq i_{t}}$ and cluster end $C_{e}=({\mathcal {E}}(i) \geq \delta)_{i \in w: i\geq i_{t}}$ positions therein, where *δ* is an integer background threshold (Figure 2A). To account for large variations in coverage between distinct genomic regions, we estimate noise levels in the coverage function at positions proximal to hcTs and use this estimate to compute a window-specific threshold *δ*_*w*_ as follows. We draw a random sample ${U} \subseteq {\mathcal {T}}$ of size *N* (here *N*=1000) and consider ${\tilde {W}}=((i_{t}-n, i_{t}+n))_{t \in U}$, i.e. a sequence of genomic intervals centered on each *i*_*t*_. By default, *n*=25. Then, we compute normalized non-zero coverage differences *D* ^+^ within ${\tilde {W}}$. Let

$$D=\left(\frac{{\mathcal S}({\tilde w})}{\text{max}({\mathcal S}({\tilde w}), {\mathcal E}({\tilde w}))}, \frac{{\mathcal E}({\tilde w})}{\text{max}({\mathcal S}({\tilde w}), {\mathcal E}({\tilde w}))} \right)_{{\tilde w} \in {\tilde W}} $$

be the sequence of all normalized coverage fluctuations observed within $\tilde W$. We model the distribution of nonzero fluctuations *D* ^+^ as a mixture of two Gaussian components with unequal variance. The first component (*k*=1) models noisy fluctuations, while the second component (*k*=2) models sharp jumps in the coverage function. Model parameters are estimated using expectation-maximization and responsibilities are used to compute the coefficient *c*= min{*x*∈*D* ^+^:*p*(*k*=2|*x*)≥*p*(*k*=1|*x*)}, which along with $\text {max}({\mathcal S}(w))$ and $\text {max}({\mathcal E}(w))$ determines *δ*_*w*_ within each window. Alternatively, the user can define a global threshold, e.g. by selecting a fraction of the minimum coverage requirement *m*_1_ at hcTs or of the mode *m*_2_ of the coverage distribution at these sites. A choice *δ*=⌊0.1·max(*m*_1_,*m*_2_)⌋, where ⌊*x*⌋ is the largest integer not greater than *x*, empirically works well on all analyzed PAR-CLIP datasets.

#### Identify candidate cluster boundaries

The width $W=(l-k+1)_{(k,l) \in C_{s} \times C_{e}}$ of each candidate cluster and signal levels at *C*_*s*_ and *C*_*e*_, namely $n_{s}=({\mathcal S}(i))_{i \in C_{s}}$ and $n_{e}=({\mathcal E}(i))_{i \in C_{e}}$, respectively, are computed (Figure 2A).

#### Represent candidate clusters as rank vectors

We represent each candidate cluster characterized by the vector $(n_{s_{k}}, n_{e_{l}}, w_{\textit {kl}})$ as a rank vector $\phantom {\dot {i}\!}\mathbf r_{\textit {kl}}=(r_{s_{k}}, r_{e_{l}}, r_{w_{\textit {kl}}})$ (Figure 2A), where *r*_*s*_ is the ranking of start positions (with ties resolved 5’ →3’, i.e. with increasing values at each index set of ties), *r*_*e*_=*π*(*n*_*e*_) is the ranking of end positions (with ties resolved 3’ →5’) and *r*_*w*_=*π*(−*W*) is the ranking of candidate cluster widths (with ties resolved by minimum ranking).

#### Identify the optimal solution

The expected coverage of a RBP binding site corresponds to a sharply peaked rectangle function [12], represented by the rank vector *O*=(0,0,0). We identify the optimal solution as the candidate cluster that is closest to *O* in terms of the euclidean norm of its rank vector **r**_*kl*_. Although multiple optimal solutions can occur, the choice of the euclidean norm strongly disfavors large clusters by construction. Therefore, in case of ties, the shortest cluster is reported, as it corresponds to the binding site with higher signal at the cluster boundaries as compared to any other optimal solution.

### Comparison with PARalyzer

#### Data processing

All PAR-CLIP data sets were processed as previously described [11]. Briefly, adapter sequences were removed. Reads of length ≥15 passing the Illumina quality filter were aligned to the human reference assembly ‘hg19’ using Bowtie [16], allowing at most one mismatch. The following Bowtie parameters were specified: -best -chunkmbs 512 -n 1 -S -M 100.

#### Parametrization

While our method depends on few parameters - essentially the minimum required coverage at transitions and the posterior probability cutoff - PARalyzer contains a more extensive parametrization. To allow for a fair comparison we selected mainly default and recommended parameter values. Both methods differ on what constitutes the minimum required evidence for a binding site. While wavClusteR poses a cutoff *c* on the strand-specific coverage at hcTs, PARalyzer applies a threshold on the number of reads forming a read group. To compare the performance of the two methods, we first learn the PAR-CLIP specific RSF interval by fitting the mixture model using *c*=20 and then exhaustively identify binding sites with wavClusteR starting from hcTs with RSF values within the Bayes classifier and *c*=1. We run PARalyzer using two different values for the minimum conversion locations for clusters *n*, namely *n*=1 (default value) or *n*=2 (recommended value), respectively. The choice of this parameter crucially determines sensitivity and recall of the algorithm. The full set of PARalyzer parameters used for the comparison is provided in Additional file 1, Section 1.1. Only clusters exhibiting at least one T to C transition with a strand-specific coverage of 10 are retained for the comparison, with no requirement on its RSF value to enable a fair comparison between algorithms.

#### microRNA seed mapping

We considered a set of microRNAs (miRNAs) previously shown to be expressed in HEK293 cells [17] and computed the enrichment of miRNA seeds within each set of cluster sequences relative to a random control. The latter was obtained by generating 10^4^ samples of dinucleotide shuffled microRNA sequences and the mean relative seed count was used as background estimate. To allow for a fair comparison PARalyzer-specific clusters were extended to the median length of wavClusteR-specific clusters.

### Computing false discovery rates

To provide experimentally-based estimates of the False Discovery Rate (FDR) of our method, we analyze the MOV10 PAR-CLIP data set and a matched total RNA-Seq profile from the same HEK293 cells used to perform the PAR-CLIP experiment [11].

#### FDR of high-confidence interaction sites

We estimated a highly conservative FDR upper bound and a FDR lower bound as a function of the RSF as follows. Let  be the set of genomic positions with a minimum coverage of 20 within the PAR-CLIP and the RNA-Seq data set and at least one transition within PAR-CLIP. Each element of  is associated with a specific PAR-CLIP RSF value. We partition the RSF interval (0,1] into ten equally spaced intervals and for each range we identify the genomic positions $\mathcal {P} \subseteq \mathcal {G}$ such that the associated RSF values fall into the RSF interval. We compute a conservative FDR upper bound by regarding as FPs all genomic positions $\mathcal {U} \subseteq \mathcal {P}$ showing at least one transition in the RNA-Seq data, irrespective of their RSF values. The FDR upper bound is therefore $|{\mathcal U}|/|{\mathcal P}|$. Similarly, we compute the FDR lower bound by considering FPs all genomic positions $\mathcal {L} \subseteq \mathcal {P}$ exhibiting an RNA-Seq-based RSF within the same interval, and compute the FDR lower bound as $|{\mathcal L}|/|{\mathcal P}|$.

#### FDR clusters

We rank clusters by decreasing values of relative log-odds and consider the resulting top *n* clusters. For each cluster in the ranking, we identify the set  of genomic positions with hcTs localizing therein and compute the RNA-Seq-based RSF $x_{t},t\in \mathcal {T}$. To compute conservative FDR estimates, we regard a cluster as FP if there exist at least one $t\in \mathcal {T}$ such that *a*≤*x*_*t*_≤*b*, where [ *a*,*b*] is the PAR-CLIP-specific RSF support resulting from applying a given posterior probability cutoff. This condition is highly conservative, as a single true hcT within a cluster with multiple detected hcT suffices to correctly identify the binding site. Similarly, we compute less conservative FDR values by regarding a binding site as FP if every $t\in \mathcal {T}$ satisfies *a*≤*x*_*t*_≤*b*.

## Implementation

The algorithms described above are implemented in version 2.0 of our R package wavClusteR [11]. The MRN algorithm is implemented using parallelization, as binding sites are independent of each other. A graphical outline of the data analysis workflow offered by wavClusteR is illustrated in Additional file 1, 2.1.

## Results and discussion

First, we show that the MRN algorithm provides sensitive and highly resolved identification of clusters. We then apply our method to published PAR-CLIP data sets and demonstrate part of our post-processing pipeline. We compare our algorithm to PARalyzer [13] using published AGO2 PAR-CLIP data. Finally, we report estimates of FDRs of high-confidence transitions (hcTs) and of inferred protein binding sites by integrating matched RNA-Seq data.

### Sensitive delineation of clusters at high resolution

We previously proposed an algorithm to resolve cluster boundaries by computing the continuous wavelet transform (CWT) of the coverage function around hcTs. However, this method is prone to false negatives, i.e. hcTs that are not assigned to a cluster, when genomic regions with complex coverage geometry and high variance of local signal-to-noise ratios are encountered. To address this issue and increase the sensitivity of our peak calling procedure, we developed a CWT-independent algorithm which we termed mini-rank norm (MRN). The MRN algorithm (see Methods and Figure 2) solves an optimization problem in which hcTs are first employed to reduce the search space. Signal and noise in the coverage function are locally separated by modeling coverage fluctuations and integrating knowledge of the geometric properties of RBP binding sites. By assuming that the expected coverage of a cluster corresponds to a sharply peaked rectangle function [12], all candidate cluster boundaries spanning a high-confidence PAR-CLIP signal are then exhaustively evaluated and ranked accounting for this prior knowledge. By design, our algorithm favors sharp boundaries and short cluster widths, and, thus, accurately resolves clusters even when multiple binding sites localize within close proximity (Figure 2B). In order to test whether the highly volatile coverage function of PAR-CLIP data reflects complex RBP binding profiles or is an artifact of the procedure, we analyzed published AGO2 PAR-CLIP data [18] for which we can readily evaluate identified binding sites by considering expressed microRNA sequences. Our sequence analysis of 3’-UTRs exhibiting multiple clusters resulted in a large number of transcripts (*n*=928). Each cluster localized within the 3’-UTR could be assigned to one or more microRNA seed sequences of microRNAs expressed in HEK293 cells, suggesting that these clusters correspond to biologically relevant AGO2 binding sites. Two exemplary regions are illustrated in Figure 2C-D. In addition, our hcT-centered strategy resulted on average in a ∼10x speed up over the CWT-based peak calling on all tested PAR-CLIP data sets (Additional file 1, 1.2).

### Application to published PAR-CLIP data sets

In order to place the binding preference of the RBP within the biological context, post-processing of identified binding sites is required. PARalyzer returns all identified clusters and read groups, and optionally seed-matches for supplied microRNA sequences within the resulting clusters as text files. In contrast, wavClusteR makes use of the R environment to provide extensive post-processing functions supporting i) export of the coverage function, hcTs and clusters for visualization in the UCSC genome browser; ii) export of cluster sequences in FASTA format for de novo motif discovery and motif analysis; iii) strand-specific cluster annotation across different functional transcriptome compartments in sense and antisense orientations, including normalization of observed frequencies to the overall compartment length and iv) generation of metagene profiles of clusters and their statistics to assess the protein-specific distribution of binding sites across genes. Furthermore, most BioConductor packages can directly use R objects returned by wavClusteR as an input.

**Figure 3:**
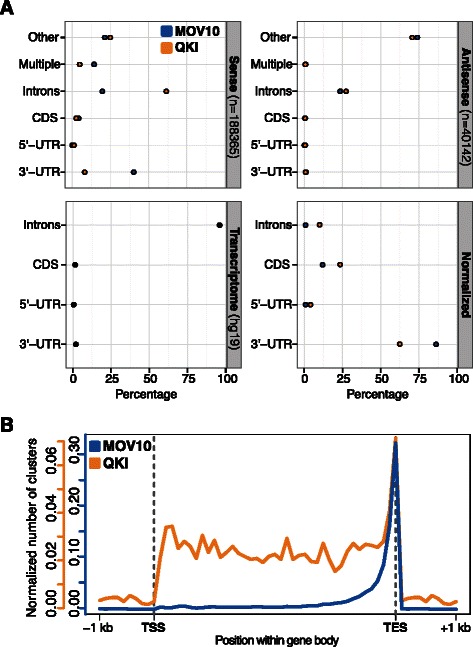
**Post-processing of binding sites identified in the MOV10 and QKI data sets.** **(A)** Annotation of MOV10 and QKI clusters with respect to the sense and antisense strand, respectively (top). The distribution of different transcript features in the human transcriptome (hg19, bottom left) is used to compute the normalized annotation profile for clusters mapping on the sense strand (bottom right). **(B)** Corresponding metagene profiles of MOV10 and QKI clusters.

For illustration, we provide examples of cluster annotations and metagene profiles obtained from PAR-CLIP data sets of MOV10 and QKI, which are characterized by different binding preferences. Annotation of MOV10 clusters shows that MOV10 preferentially binds to 3’-UTRs of transcripts [11] (Figure 3A), whereas binding sites of QKI, which regulates pre-mRNA splicing, mRNA export and stability, and protein translation [19], are enriched in 3’-UTRs, coding sequences and introns (Figure 3A). Notably, the distinct binding preferences of the two proteins are neatly reflected in their metagene profiles (Figure 3B).

### Comparison with PARalyzer

**Figure 4:**
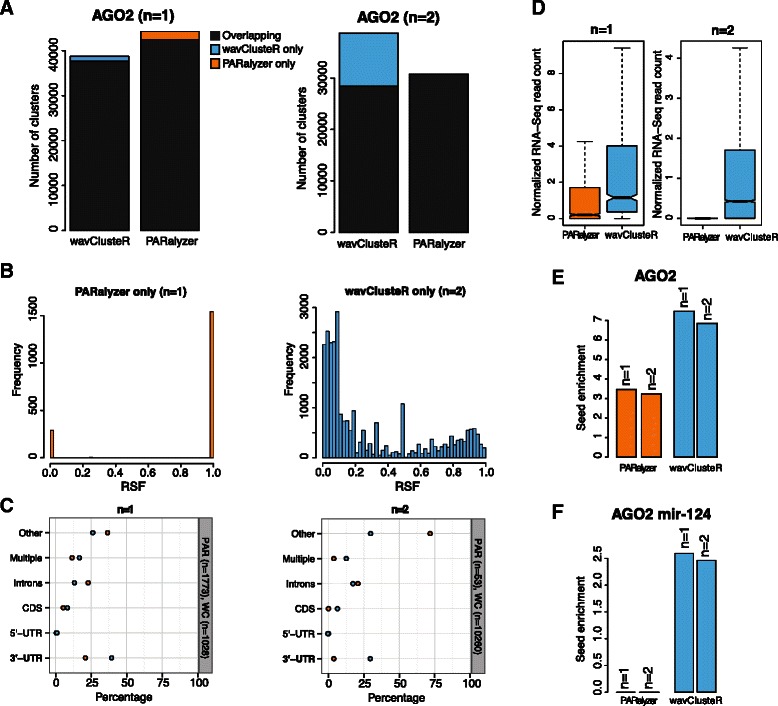
**Comparison of wavClusteR with PARalyzer on publicly available PAR-CLIP data.** **(A)** Barplot representing overlapping or exclusive clusters identified by PARalyzer (with parameters *n*=1 (left) and *n*=2 (right)) and wavClusteR on AGO2 PAR-CLIP data. Note that the number of overlapping clusters called by either method differ as there is no one-to-one correspondence between them. **(B)** Distribution of RSF values for *T*→ C transitions localizing within clusters exclusively identified by PARalyzer (*n*=1) and wavClusteR (*n*=2). **(C)** Annotation of method-specific clusters to distinct features of the human transcriptome. Blue and orange circles denote clusters exclusively identified by wavClusteR and PARalyzer, respectively. **(D)** Normalized number of RNA-Seq reads from transcripts overlapping with clusters exclusively identified by by either method. **(E)** Seed enrichments over randomized background for microRNA seeds of microRNA expressed in HEK293 cells within clusters exclusively identified by PARalyzer and wavClusteR. **(F)** Same as E, but for the mir-124 seed in AGO2 PAR-CLIP from cells transfected with this microRNA.

Using published AGO2 PAR-CLIP data sets, we compared the performance of wavClusteR with PARalyzer [13]. Our comparison revealed that the largest fraction of clusters is similarly identified by both methods (Figure 4A, see Additional file 1, 2.2 for cluster size distributions). However, depending on the parameter settings, the clusters specifically called by either method can represent a substantial fraction. Therefore, we decided to analyze method-specific clusters in more detail. The distribution of RSF values within these clusters revealed that PARalyzer-specific clusters contained almost exclusively extreme RSF values. These values are unlikely to be caused by experimental induction, as the PAR-CLIP-specific enrichment of T to C transitions is missing when compared with other substitutions exhibiting similar RSF values (Additional file 1, 2.3). In contrast, wavClusteR-specific clusters covered the entire RSF range (Figure 4B and Additional file 1, 2.4) and mostly localized within the high-confidence RSF support. In addition, analysis of the read count distribution of PARalyzer-specific clusters (Additional file 1, 2.5) ruled out that the observed extreme RSF values result from clusters with low read count, which could be otherwise filtered out using more stringent parameter cutoffs. Annotation of clusters to the transcriptome shows that PARalyzer-specific AGO2 clusters preferentially localize within intergenic regions or introns (Figure 4C). In contrast, wavClusteR-specific binding sites mainly fall into 3’UTRs, which agrees well with the known biological function of the AGO2 protein [20,21]. Furthermore, we integrated RNA-Seq data derived from the same cell line to independently assess expression of the identified clusters. PARalyzer-specific clusters show significantly reduced expression levels (Figure 4D). This analysis suggests that the largest proportion of PARalyzer-specific clusters corresponds to false positives, possibly caused by RNA contamination during the experimental procedure, as most of the cluster-containing transcripts show no detectable expression.

An additional criterion to determine whether an AGO2 cluster, identified by either PARalyzer or wavClusteR, corresponds to a *bona fide* binding site is whether the site can be assigned to any expressed miRNA. Therefore, we decided to evaluate the quality of the method-specific clusters by considering the presence of seeds of miRNAs known to be expressed in HEK293 cells. Since miRNAs target AGO2 proteins by complementary base pairing [20], we searched for corresponding seed sequences within the identified AGO2 binding sites (see Methods). Our analysis revealed that wavClusteR-specific clusters were substantially more enriched (>2 folds) for miRNA seeds than PARalyzer-specific ones, suggesting that these clusters more accurately reflect AGO2 binding sites. In addition, we repeated the analyses using PAR-CLIP datasets from mir-124 miRNA transfection experiments [8] to quantify the fraction of the PARalyzer- and wavClusteR-specific clusters that could be assigned to the transfected miRNA. Figure 4F shows an enrichment of mir-124 seeds within wavClusteR-specific clusters, which is missing in clusters exclusively called by PARalyzer. Finally, these results are further supported by the analysis of a previously published Pumilio-2 (PUM2) PAR-CLIP data set [8]. This RNA-binding protein recognizes a well characterized UGUAHAUA motif [22], which we found to be strongly enriched in wavClusteR-specific clusters (20.2%, *n*=1777) with respect to PARalyzer-specific ones (0.9%, *n*=219 and standard parameters).

### Experimentally-based estimation of false discovery rates

**Figure 5:**
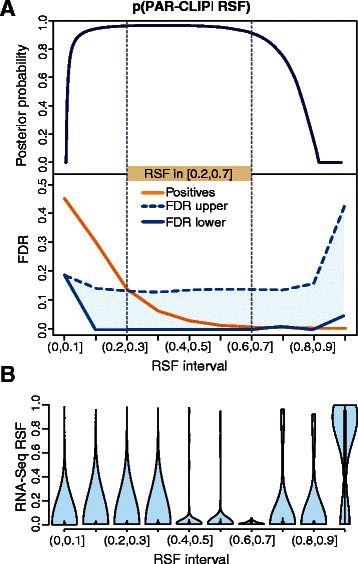
**False Discovery Rate of high-confidence transitions.** **(A)** Posterior probability of PAR-CLIP induced RSF values (top) and corresponding lower and upper bounds of the FDR (bottom) over the RSF interval. The normalized fraction of positives is shown in orange. **(B)** Distribution of RNA-Seq RSF values over the RSF interval considered above.

We assessed the FDR of our high-confidence transitions by integrating matched total RNA-Seq data from HEK293 cells [11]. We reason that no cross-linking induced transitions are present in RNA-Seq. Hence, if our model correctly identifies PAR-CLIP induced RSF value, a transition classified as PAR-CLIP-specific and equally found in RNA-Seq data is likely to correspond to a false positive (FP). We partitioned the entire RSF interval (0,1] into different subsets and used transitions identified in both PAR-CLIP and RNA-Seq data to compute a highly conservative FDR upper bound, treating all observed RNA-Seq transitions as FPs irrespective of their RSF values (see Methods). Our analysis shows that the RSF interval [0.2,0.7], which we previously reported as PAR-CLIP-specific [11], is bounded by the lowest FDRs values (Figure 5A), thus demonstrating the high precision of our approach. Furthermore, the distribution of RNA-Seq RSF values within the central partitions of the RSF interval (Figure 5B) are mainly dominated by low RSF values compatible with sequencing errors, indicating that our FDR estimates are highly conservative.

Next, we assessed the FDR of clusters, which potentially contain multiple interaction sites. We considered increasing posterior probability cutoffs *δ* and computed highly conservative FDR estimates of clusters (see Methods) obtained for each threshold (Table 1). At *δ*=0.9, our method identified 66.837 MOV10 clusters (of which 20% contained a single hcT) at a ≤3*%* FDR for the top 250 clusters ranked by relative log-odds (Table 1). Notably, the FDR values dropped substantially from *δ*=0.7 to *δ*=0.9 without a major effect on the total number of reported clusters, thus showing that stringency of the analysis can be effectively tuned by this parameter. This property is desirable for experimental validation, which is generally performed on few top ranked candidates only.

**Table 1:** **Conservative FDR estimates of clusters as a function of different posterior probability cutoffs (see** Methods **)**

**Posterior cutoff (** ***δ*** **)**	**>0.7**	**>0.8**	**>0.9**
RSF support	[0.014,0.808]	[0.021,0.779]	[0.044,0.713]
No. hcTCs	268.771	265.085	246.619
No. clusters	67.856	67.493	66.837
with 1 hcTC	13.227 (19.5%)	13.351 (19.8%)	13.471 (20.1%)
FDR top 75	0.066	0.066	0.024
FDR top 125	0.096	0.085	0.0266
FDR top 250	0.132 (0.02)	0.108 (0.02)	0.028 (0.012)

FDR values in parenthesis refer to more relaxed FDR estimates.

## Conclusion

We presented a sensitive and comprehensive framework for PAR-CLIP data analysis, which provides statistically grounded and biologically interpretable results. In our approach, not the total number of interaction sites or observed transitions are considered, but rather the frequency at which expected transitions occur. This transition-based strategy outperforms hard thresholding-based approaches and achieves higher sensitivity and specificity.

## Availability and requirements

- **Project name:** wavClusteR
- **Project home page:** https://github.com/FedericoComoglio/wavClusteR
- **Operating system(s):** Platform independent
- **Programming language:** R
- **Other requirements:** R?>?= 3.0.0
- **License:** GPL-2
- **Any restrictions to use by non-academics:** none

## Additional file

Additional file 1
**Supplementary methods and results.** PARalyzer parameters, graphical outline of wavClusteR 2.0 and analysis of publicly available PAR-CLIP data sets.
