# Supplementary material for: Sensitive and highly resolved identification of RNA-protein interaction sites in PAR-CLIP data
Source: BMC Bioinformatics. 2015 Feb 1;16:32. doi: 10.1186/s12859-015-0470-y (PMC4339748; doi:10.1186/s12859-015-0470-y)
Supplement: Additional file 1 — Supplementary methods and results. PARalyzer parameters, graphical outline of wavClusteR 2.0 and analysis of publicly available PAR-CLIP data sets. [file 12859_2015_470_MOESM1_ESM.pdf]

Supplementary Information for:
Sensitive and highly resolved identification of RNA-protein interaction sites in PAR-CLIP data

Federico Comoglio[1], Cem Sievers[1,2,3] and Renato Paro[1,4]

[1]Department of Biosystems Science and Engineering,
Swiss Federal Institute of Technology Zurich, Mattenstrasse 26, 4058 Basel, Switzerland

[2] Department of Pathology and Center for Cancer Research Massachusetts General Hospital and Harvard Medical School, Boston, Massachusetts, USA

[3] Broad Institute of MIT and Harvard, Cambridge, Massachusetts, USA

[4]Faculty of Science, University of Basel, Klingelbergstrasse 50, 4056 Basel, Switzerland

December 14, 2014

Contents

1 Supplementary Methods **1**
- 1.1 PARalyzer parameters 1
- 1.2 Analysis of publicly available PAR-CLIP data sets 2

2 Supplementary Results **2**
- 2.1 Graphical outline of wavClusteR 2
- 2.2 Distribution of cluster sizes 4
- 2.3 Distribution of substitutions in AGO2 PAR-CLIP data 4
- 2.4 Distribution of RSF values for transitions localizing within method-specific clusters 5
- 2.5 Read count distribution of PARalyzer-specific clusters 6

1 Supplementary Methods

1.1 PARalyzer parameters

The following PARalyzer parameters were provided in the initialization (`.ini`) file to carry out the $n = 1$ analysis:

```
BANDWIDTH=3
CONVERSION=T>C
MINIMUM_READ_COUNT_PER_GROUP=10
MINIMUM_READ_COUNT_PER_CLUSTER=1
MINIMUM_READ_COUNT_FOR_KDE=5
MINIMUM_CLUSTER_SIZE=1
MINIMUM_CONVERSION_LOCATIONS_FOR_CLUSTER=1
MINIMUM_CONVERSION_COUNT_FOR_CLUSTER=1
MINIMUM_READ_COUNT_FOR_CLUSTER_INCLUSION=1
MINIMUM_READ_LENGTH=1
MAXIMUM_NUMBER_OF_NON_CONVERSION_MISMATCHES=5
ADDITIONAL_NUCLEOTIDES_BEYOND_SIGNAL=0
```

Whereas to perform the $n = 2$ analysis the following parameters were specified:

```
BANDWIDTH=3
CONVERSION=T>C
MINIMUM_READ_COUNT_PER_GROUP=10
MINIMUM_READ_COUNT_PER_CLUSTER=1
MINIMUM_READ_COUNT_FOR_KDE=5
MINIMUM_CLUSTER_SIZE=1
MINIMUM_CONVERSION_LOCATIONS_FOR_CLUSTER=2
MINIMUM_CONVERSION_COUNT_FOR_CLUSTER=1
MINIMUM_READ_COUNT_FOR_CLUSTER_INCLUSION=1
MINIMUM_READ_LENGTH=1
MAXIMUM_NUMBER_OF_NON_CONVERSION_MISMATCHES=5
ADDITIONAL_NUCLEOTIDES_BEYOND_SIGNAL=0
```

1.2 Analysis of publicly available PAR-CLIP data sets

We applied our method to the analysis of the published PAR-CLIP data sets indicated in Table 1.

Dataset	GEO accession no.	Reference	no. of aligned reads ($\cdot 10^6$)
**AGO2**	GSM714644 and -45	[Kishore *et al.*, 2011]	11.67
**AGO2-mir124**	GSM545216	[Hafner *et al.*, 2010]	1.73
**MOV10**	GSM921128	[Sievers *et al.*, 2012]	9.08
**PUM2**	GSM545210	[Hafner *et al.*, 2010]	1.61
**QKI**	GSM545211	[Hafner *et al.*, 2010]	2.90

Table 1: Summary of publicly available PAR-CLIP data sets used to assess the performance of wavClusteR and for the comparison with PARalyzer.

2 Supplementary Results

2.1 Graphical outline of wavClusteR

The wavClusteR package, version 2.0, is composed of 19 main functions, 7 of which constitute the package core, 8 return a graphical output and 4 can be used to export raw and processed data to the UCSC genome browser for visualization. A graphical representation of the data analysis workflow offered by the package, illustrating its functions and parameters, is provided in Figure 5.

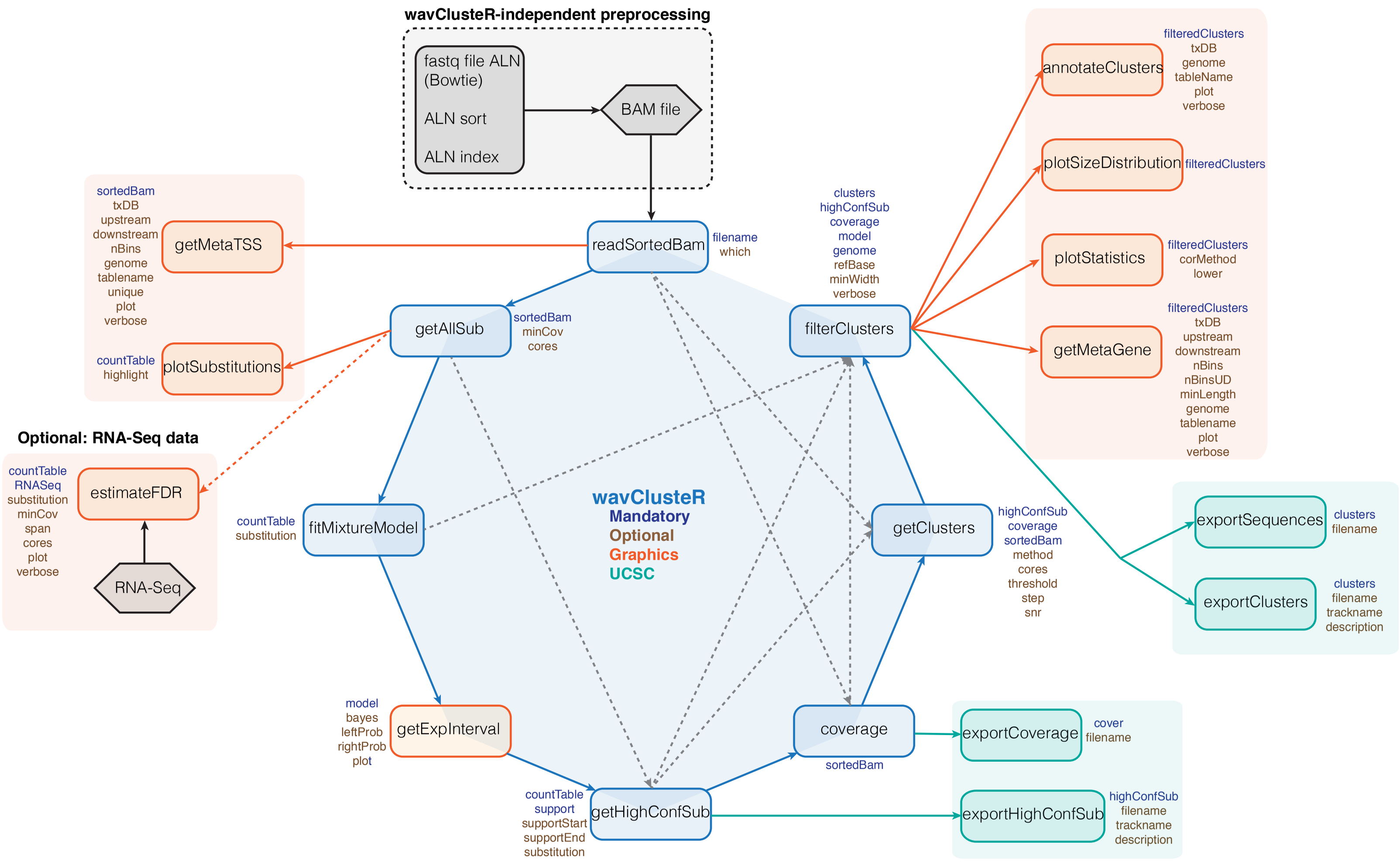

Figure 1: Schematic representation of wavClusteR functions (rectangles) and their dependencies (arrows). Mandatory and optional parameters are indicated in blue and brown, respectively. The analysis requires a sorted and indexed BAM file (hexagon, top). The package core functions (blue) perform a memory-efficient import of this file, extract all base substitutions, fit the mixture model, classify transitions, compute the coverage, identify clusters and compute cluster statistics. Post-processing functions (orange) allow to analyze these clusters with respect to their biological context. Cluster sequences can be exported for motif search; data and results can be exported for visualization in the UCSC genome browser (green).

2.2 Distribution of cluster sizes

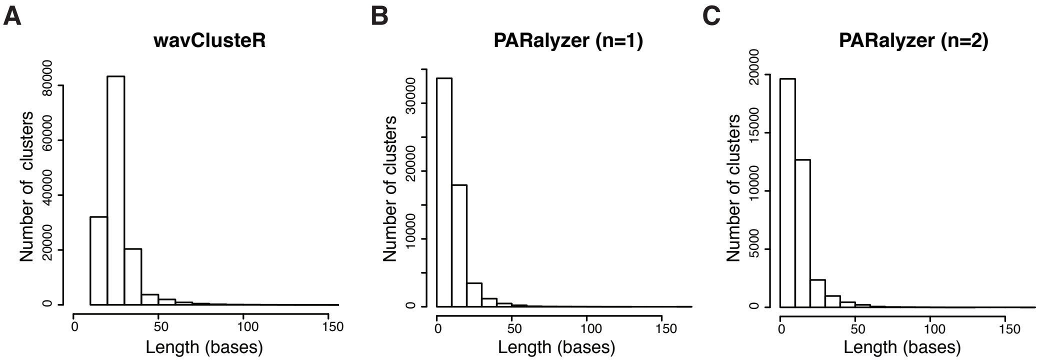

Figure 2: (A) Size distribution of clusters returned by wavClusteR. (B) Same as A, for clusters returned by PARalyzer ($n = 1$). (C) Same as A, for clusters returned by PARalyzer ($n = 2$).

2.3 Distribution of substitutions in AGO2 PAR-CLIP data

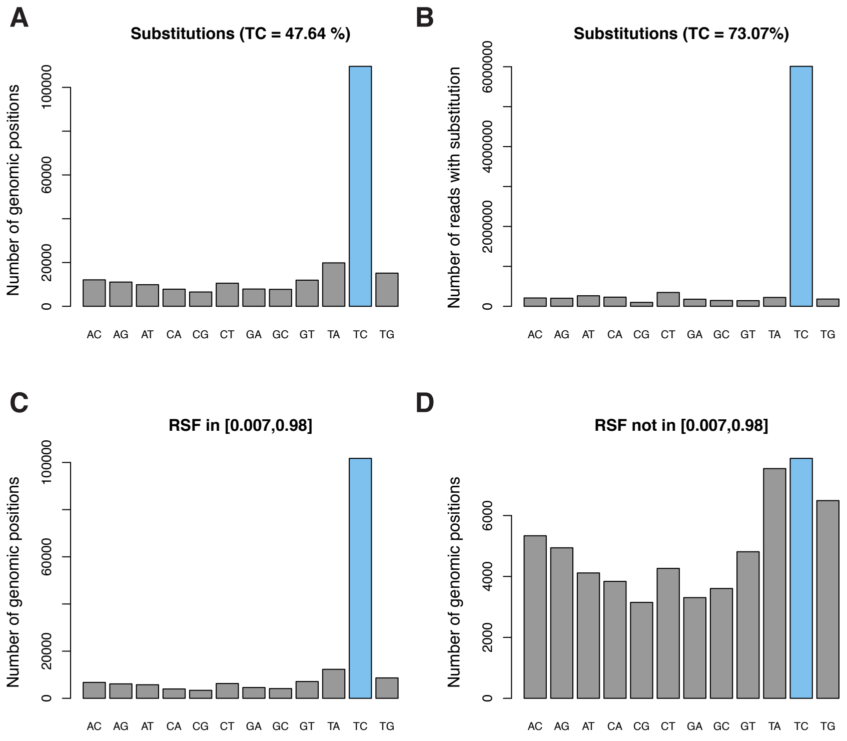

Figure 3: (A) Number of genomic sites exhibiting the indicated substitution in AGO2 PAR-CLIP data. (B) Total number of reads exhibiting the indicated substitution. (C) Same as A, but for transitions with RSF values falling within the PAR-CLIP specific RSF interval identified by our model. (D) Same as A, but for transitions with extreme RSF values.

2.4 Distribution of RSF values for transitions localizing within method-specific clusters

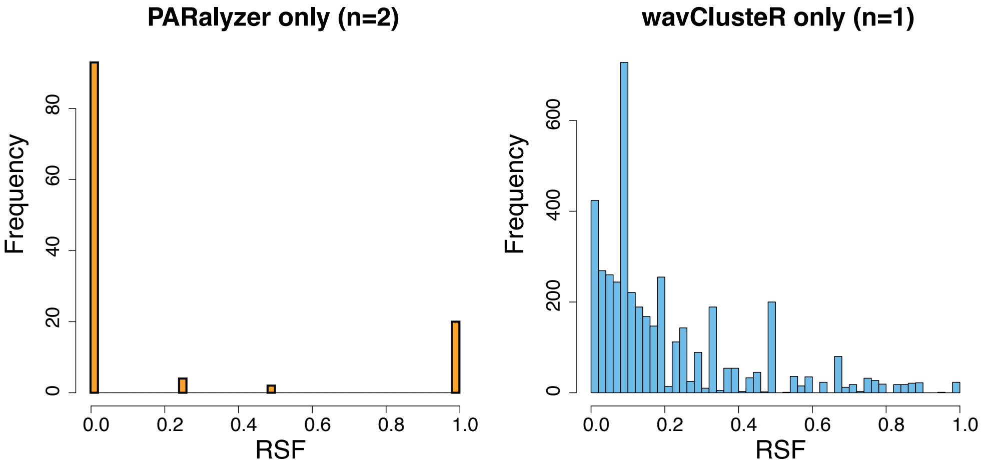

Figure 4: Distribution of RSF values for $T \rightarrow$ C transitions localizing within clusters exclusively identified by PARalyzer ($n = 2$) and wavClusteR ($n = 1$). These barplot complement Figure 4B in the main text.

2.5 Read count distribution of PARalyzer-specific clusters

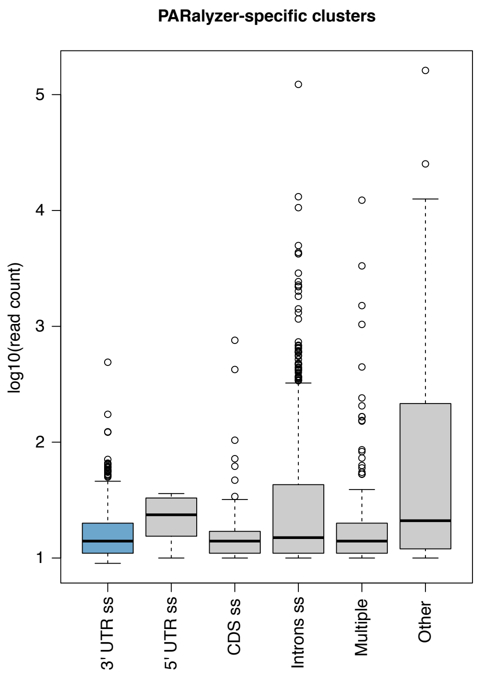

Figure 5: Read count distribution of PARalyzer-specific clusters annotating within different functional compartments.

References

[Hafner *et al.*, 2010] Hafner M., Landthaler M. *et al.* (2010) Transcriptome-wide identification of RNA-binding protein and microRNA target sites by PAR-CLIP, Cell, **141**, 129-141

[Kishore *et al.*, 2011] Kishore S., Jaskiewicz L. *et al.* (2011) A quantitative analysis of CLIP methods for identifying binding sites of RNA-binding proteins, Nat Methods, **8**, 559-564

[Sievers *et al.*, 2012] Sievers C., Schlumpf T. *et al.* (2012) Mixture models and wavelet transforms reveal high confidence RNA-protein interaction sites in MOV10 PAR-CLIP data, Nucleic Acids Res, **40**, e160
